# Low dielectric and low surface free energy flexible linear aliphatic alkoxy core bridged bisphenol cyanate ester based POSS nanocomposites

**DOI:** 10.3389/fchem.2013.00019

**Published:** 2013-10-14

**Authors:** S. Devaraju, P. Prabunathan, M. Selvi, M. Alagar

**Affiliations:** ^1^Polymer Composites Lab, Department of Chemical Engineering, Alagappa College of Technology, Anna UniversityChennai, India; ^2^Next MEMS lab, School of Mechanical Engineering, Pusan National UniversityBusan, South Korea

**Keywords:** organic-inorganic hybrid, cyanate ester, POSS, thermal properties, low-k, low surface free energy

## Abstract

The aim of the present work is to develop a new type of flexible linear aliphatic alkoxy core bridged bisphenol cyanate ester (AECE) based POSS nanocomposites for low k applications. The POSS-AECE nanocomposites were developed by incorporating varying weight percentages (0, 5, and 10 wt %) of octakis (dimethylsiloxypropylglycidylether) silsesquioxane (OG-POSS) into cyanate esters. Data from thermal and dielectric studies imply that the POSS reinforced nanocomposite exhibits higher thermal stability and low dielectric value of *k* = 2.4 (10 wt% POSS-AECE_4_) compared than those of neat AECE. From the contact angle measurement, it is inferred that, the increase in the percentage incorporation of POSS in to AECE, the values of water contact angle was enhanced. Further, the value of surface free energy was lower when compared to that of neat AECE. The molecular level dispersion of POSS into AECE was ascertained from SEM and TEM analyses.

## Introduction

High-performance dielectric materials have attracted much attention in worldwide, owing to their widespread applications in many rapidly developing fields such as electric power, micro-electrics, informatics, etc. (Phang et al., 2009; Jang et al., 2010; Zhuo et al., 2012). The hybridization of organic and inorganic components is the most effective method to develop high performance dielectrics, since hybridization merges the advantages of both organic and inorganic materials, and thus, has the biggest benefit to get the desired integrated performance. To date, many inorganic-organic hybrids have been investigated, and the results show that the properties of these inorganic-organic hybrids are greatly dependent on the nature of the inorganic and organic segments, and their interfacial adhesion (Laine, 2005; Liu et al., 2005; Zhang et al., 2005; Lee et al., 2006; Dang et al., 2008; Sanchez et al., 2011a,b).

The hydrophobic polymers have been a topic of considerable interest owing to their attractive properties such as low surface energy, heat resistance, chemical inertness, and low dielectric constant. Hydrophobic coatings may be used in various industrial applications including anti-wetting, anti-snow adherence, anti-rusting, reduced friction resistance etc. Low surface energy polymeric materials with good film-forming characteristics have attracted great interest because of their practical applications (Jeyaprakash et al., 2004; Yoshida et al., 2004; Qu et al., 2008). Consequently great attention has been placed on precise strategies modifying these solid surfaces. Most of the low surface energy polymeric materials that have been developed were based on flurorine- or silicon-containing polymers (Liang et al., 2011; Qu and Xin, 2011; Raza et al., 2012). Polyhedral oligomeric silsesquioxanes (POSS) have recently attracted considerable attention. The hybrid structure has significant contribution to dielectric, heat-resistant and radiation-resistant behavior of coatings. The POSS based hybrid coatings after radiation treatment forms the self-passive silica layer, which prevents further deterioration of the surface. An introduction of POSS in to polymers brings about significant improvements in their properties such as increased thermal stability, increased hardness and oxidative resistance, increased Tg, mechanical properties and decreased flammability and viscosity during processing, increase in hydrophobicity and wear resistance as well as decrease in friction on the surfaces of the POSS-based nanocomposites (Gonzalez et al., 2000; Devaux et al., 2002; Xu et al., 2002; Leu et al., 2003; Wu et al., 2007; Tang and Lewin, 2009; Cordes et al., 2010; Nagendiran et al., 2010; Devaraju et al., 2011, 2012; Kuo and Chang, 2011; Venkatesan et al., 2011, 2012; Chandramohan et al., 2012; Zhang and Müller, 2013).

Cyanate ester (CE) resins have outstanding integrated properties, such as excellent dielectric value, thermal resistance as well as good processing characteristics etc., and hence CE resins have been regarded as potential candidates with the greatest competition to fabricate advanced functional, structural composite materials for many cutting edge fields including microelectronics, aerospace, and transportation (Fang and Shimp, 1995; Zhuo et al., 2011). CE resins are superior to conventional epoxy, polyimide, and BMI resins. For example, the moisture absorption rate of cyanate esters is lower than that of epoxy, polyimide, and BMI resins. Cyanate esters undergo thermal or catalytic cyclotrimerization to form triazine rings during curing. However, the major drawback of CE resin is its inherent brittleness, which often restricts its structural applications. In addition, CE needs to be cured at very high temperatures and the resins cured at high temperature tend to have higher stress concentration and internal defects, and thereby declining the integrated properties of the resultant materials in addition to high energy consumption.

In the present work, an attempt has been made to develop a series of flexible linear aliphatic alkoxy core bridged bisphenol cyanate esters based OG-POSS nanocomposites. The newly synthesized linear aliphatic alkoxy core bridged bisphenol CE monomers exhibit lower curing temperature than that of conventional CE (BADy). The OG-POSS was introduced into cyanate esters in varying weight percentages (5 and 10 wt %), and their thermal, dielectric and morphological properties were studied.

## Materials

Cyanogen bromide, dibromoalkane (1,2-dibromoethane, 1,4-dibromobutane, 1,6-dibromohexane, and 1,8-dibromooctane) were purchased from Spectrochem, India. 3-chloroperoxybenzoic acid (MCPBA) was purchased from Sigma Aldrich, India. 4-hydroxy benzaldehyde, triethylamine, sodium carbonate, sodium sulfate, sodium hydroxide, and other solvents were obtained from SRL India and were used without further purification. OG-POSS was synthesized as per the procedure reported (Chandramohan et al., 2012; Venkatesan et al., 2012).

### Synthesis of 4, 4′-[alkane-1,2-diylbis(oxy)] dibenzaldehyde (AEDA)

**Scheme 1:**
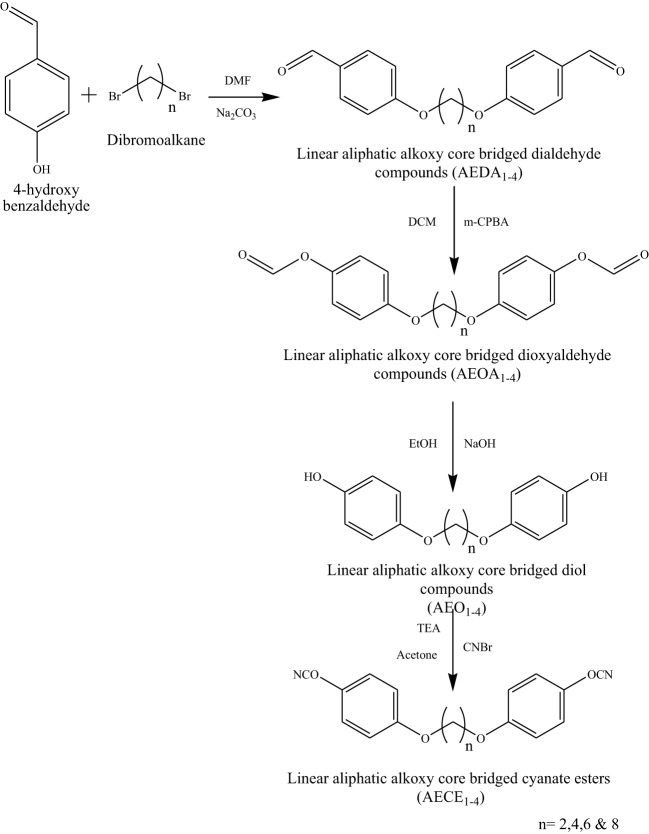
**Synthesis of linear aliphatic alkoxy core bridged cyanate ester monomers (AECE1-4)**.

^1^HNMR: (300 MHz, CDCl3) δ (ppm) and ^13^CNMR: δ(ppm) for

A two-necked round-bottomed flask, equipped with a magnetic stirrer bar, thermometer, and condenser was charged with 4-hydroxybenzaldehyde (0.0819 mol), dibromoalkane (0.0409 mol), Na_2_CO_3_ (0.1638 mol), and DMF (50 ml). The mixture was stirred and heated to reflux (140°C, 6 h). The reaction mixture was cooled to room temperature and quenched with distilled water and then filtered and washed with water to obtain a crude product. An off-white solid was obtained by recrystallization in ethanol, and the product was collected by filtration and dried at 50°C under vacuum in a hot air oven for 6 h (Scheme 1).

**AEDA_1_:** 9.9 (s, 2 H, Ar-CHO-), 7.8 (d, 4 H, ArH), 7.0 (d, 4 H, ArH), 4.5 (t, 4 H, Ar-O-CH_2_) and 190, 164, 132, 129, 115 (aromatic carbon), 68(aliphatic carbon).

**AEDA_2_:** 9.8 (s, 2 H, Ar-CHO-), 7.8 (d, 4 H, ArH), 6.9 (d, 4H, ArH), 4.1 (t, 4H, Ar-O-CH_2_), 2.0 (m, 4H, CH_2_-CH_2_) and 191, 164, 132, 130, 114 (aromatic carbon), 67, 26 (aliphatic carbon).

**AEDA_3_:** 9.9 (s, 2H, Ar-CHO-), 7.8 (d, 4H, ArH), 7.0 (d, 4H, ArH), 4.1 (t, 4H, Ar-O-CH_2_), 1.5-1.9 (m, 8H, CH_2_-CH_2_) and 190, 163, 131, 129, 115 (aromatic carbon), 67, 28, 25 (aliphatic carbon).

**AEDA_4_:** 9.8 (s, 2H, Ar-CHO-), 7.8 (d, 4H, ArH), 6.9 (d, 4H, ArH), 4.0 (t, 4H, Ar-O-CH_2_), 1.4-1.9 (m, 12H, CH_2_-CH_2_) and 191, 163, 132, 129, 115 (aromatic carbon), 68, 27, 25 (aliphatic carbon).

### Synthesis of 4, 4′-[alkane-1,2-diylbis(oxy-1,1-phenylene)]-diformate (AEOA)

A two-necked round-bottomed flask, equipped with a magnetic stirrer bar, thermometer, and condenser was charged with 4,4′-[alkane-1,2-diylbis(oxy)] dibenzaldehyde (0.0370 mol) and 3-chloroperoxybenzoic acid (0.1480 mol) was added portion wise in DCM (100 ml). The mixture was stirred at room temperature for 4 h. A milky white suspension was formed, and the resultant reaction mixture was treated with saturated NaHCO_3_ solution (50 ml). After 2 h stirring, the mixture was separated, the DCM layer was collected separately, and the aqueous layer was washed with DCM (3 × 10 ml). The organic layers were combined and washed with 10% Na_2_S_2_O_3_ solution (3 × 10 ml), followed by water (3 × 10 ml) and brine solution (3 × 10 ml), and finally dried over anhydrous sodium sulfate. The solvent was removed by rotary evaporation under reduced pressure to obtain a crude product. A white solid was obtained by recrystallization from warm ethanol, and the product was collected by filtration and dried in a desiccator (Scheme 1).

^1^HNMR: (300 MHz, CDCl_3_) δ (ppm) and ^13^CNMR: δ (ppm) for:

**AEOA_1_**: 8.3 (s, 2H, Ar-OCHO-), 7.0 (d, 4H, ArH), 6.8 (d, 4H, ArH), 4.3 (t, 4H, Ar-O-CH_2_) and 160, 157, 143, 121, 115 (aromatic carbon), 68 (aliphatic carbon).

**AEOA_2_**: 8.2 (s, 2H, Ar-OCHO-), 7.0 (d, 4H, ArH), 6.9 (d, 4H, ArH), 4.0 (t, 4H, Ar-O-CH_2_), 1.9 (m, 4H, CH_2_-CH_2_) and 159, 157, 143, 121, 115 (aromatic carbon), 68, 28 (aliphatic carbon).

**AEOA_3_:** 8.3 (s, 2H, Ar-OCHO-), 7.1 (d, 4H, ArH), 7.0 (d, 4H, ArH), 4.0 (t, 4H, Ar-O-CH_2_), 1.5-1.9 (m, 8H, CH_2_-CH_2_) and 160, 157, 143, 121, 114 (aromatic carbon), 68, 28, 26 (aliphatic carbon).

**AEOA_4_:** 8.2 (s, 2H, Ar-OCHO-), 7.0 (d, 4H, ArH), 6.7 (d, 4H, ArH), 3.9 (t, 4H, Ar-O-CH_2_), 1.3-1.8 (m, 12H, CH_2_-CH_2_) and 159, 157, 143, 121, 115 (aromatic carbon), 68, 29, 25 (aliphatic carbon).

### Synthesis of 4, 4′-[alkane-1,2-diylbis(oxy)]diphenol (AEO)

A two-necked round-bottomed flask, equipped with a magnetic stirrer bar, thermometer, and condenser was charged with 4, 4′-[Alkane-1,2-diylbis(oxy-1,1-phenylene)]-diformate (0.0331 mol), sodium hydroxide (0.1323 mol), ethanol (80 ml), and water (30 ml). The mixture was stirred and heated to reflux (110°C, 48 h). The reaction mixture was cooled to room temperature and neutralized with hydrochloric acid (3 M), forming a solid that was extracted into the DCM. The DCM layers were combined, washed with water and dried over sodium sulfate; the solvent was removed by rotary evaporation under reduced pressure to obtain a crude product. A solid was obtained by recrystallization in warm ethanol; then, it was collected by filtration and dried in a hot air oven at 50°C for 6 h, and stored in a desiccator (Scheme 1).

^1^HNMR: (300 MHz, CDCl_3_) δ (ppm) and ^13^CNMR: δ (ppm) for:

**AEO_1_:** 6.7-6.6 (d, 4H, Ar-H), 4.2 (t, 4H, Ar-O-CH_2_) and 151, 150, 115, 114 (aromatic carbon), 68 (aliphatic carbon).

**AEO_2_:** 6.7-6.6 (d, 4H, Ar-H), 4.0 (t, 4H, Ar-O-CH_2_), 1.9 (m, 4H, -CH_2_-CH_2_) and 151, 150, 115, 114 (aromatic carbon), 68, 29 (aliphatic carbon).

**AEO_3_:** 6.7-6.5 (d, 4H, Ar-H), 3.9 (t, 4H, Ar-O-CH_2_), 1.4-1.8 (m, 8H, -CH_2_-CH_2_) and 151, 150, 115, 114 (aromatic carbon), 68, 29, 26 (aliphatic carbon).

**AEO_4_:** 6.7-6.6 (d, 4H, Ar-H), 4.0 (t, 4H, Ar-O-CH_2_), 1.3-1.9 (m, 12H, -CH_2_-CH_2_) and 152, 151, 115, 114 (aromatic carbon), 68, 29, 25 (aliphatic carbon).

### Synthesis of 4, 4′-[alkane-1,2-diylbis(oxy-1,1-phenylene)]dicyanate (AECE)

A three-necked round-bottomed flask, equipped with a magnetic stirrer bar, a dropping funnel, and a drying tube, was charged with 4, 4′-[Alkane-1,2-diylbis(oxy)]diphenol (0.0203 mol) and cyanogen bromide (0.0447 mol). The reactants were dissolved in acetone (75 ml), and the solution was cooled to below −5°C in a salt ice bath. Triethylamine (0.0507 mol) was added dropwise via the dropping funnel to the reaction mixture and was maintained below −5°C. After the addition of triethylamine, the reaction mixture was left stirring for 1 h and then allowed to 25°C. The mixture was washed by fast stirring with water to remove triethylamine hydrobromide (byproduct) and the remaining precipitate was filtered and washed with water. A light white solid product was obtained by recrystallization from 1:1 mixture of ethanol and water (Scheme 1).

**AECE_1_:**

FT-IR: (KBr, cm^−1^): 2937(symmetric stretching), 2862(asymmetric stretching) for CH_2_ aliphatic Stretching, 2277, 2229 (-OCN stretching), 1243(Ar-O-CH_2_).

^1^HNMR: (300 MHz, CDCl_3_) δ (ppm): 7.2 (d, 4 H, ArH), 7.0 (d, 4 H, ArH), 4.3 (t, 4H, Ar-O-CH_2_).

^13^CNMR: δ (ppm): 157,146,116,115, 109 (aromatic carbon), 68(aliphatic carbon).

**AECE_2_:**

FT-IR: (KBr, cm^−1^): 2940 (symmetric stretching), 2858 (asymmetric stretching) for CH_2_ aliphatic Stretching and 2278, 2233 (-OCN stretching), 1249 (Ar-O-CH_2_).

^1^HNMR: (300 MHz, CDCl_3_) δ (ppm): 7.2 (d, 4 H, ArH), 6.9 (d, 4 H, ArH), 4.0 (t, 4 H, Ar-O-CH_2_), 2.0 (m, 4 H, -CH_2_-).

^13^CNMR: δ (ppm): 157,146,116, 115,109 (aromatic carbon), 68, 25 (aliphatic carbon).

**AECE_3_:**

FT-IR: (KBr, cm^−1^): 2941 (symmetric stretching), 2862 (asymmetric stretching), 2272, 2230 (-OCN stretching) for CH_2_ aliphatic Stretching, 1245(Ar-O-CH_2_).

^1^HNMR: (300 MHz, CDCl_3_) δ (ppm): 7.2 (d, 4 H, ArH), 6.9 (d, 4 H, ArH), 4.0 (t, 4 H, Ar-O-CH_2_), 1.8 (m, 4H, -CH_2_-), 1.5(m, 4H, -CH_2_-).

^13^CNMR: δ (ppm): 157,146,116,115,109 (aromatic carbon), 68, 29, 25, (aliphatic carbon).

**AECE_4_:**

FT-IR: (KBr, cm^−1^): 2937(symmetric stretching), 2856 (asymmetric stretching) for CH_2_ aliphatic Stretching, 2278, 2230 (-OCN stretching), 1242 (Ar-O-CH_2_).

^1^HNMR: (300 MHz, CDCl_3_) δ (ppm): 7.2 (d, 4 H, ArH), 6.8 (d, 4 H, ArH), 4.0 (t, 4 H, Ar-O-CH_2_), 1.9-1.7 (m, 8 H, -CH_2_-), 1.4 (m, 4 H, -CH_2_-).

^13^CNMR: δ (ppm): 156,145,115,114, 107 (aromatic carbon), 67, 27, 21 (aliphatic carbon).

### Preparation of POSS-CE nanocomposites

**Scheme 2:**
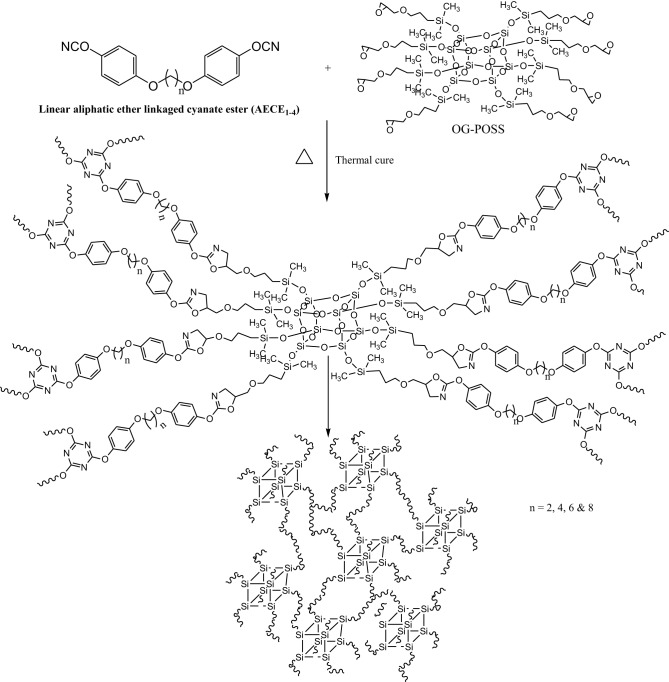
**Schematic representation of POSS-AECE nanocomposites**.

2 g of CE monomer was first dissolved in 10 ml of 1,4-dioxane. The desired amount of OG-POSS (0, 5, and 10 wt %) was dissolved in minimum amount of 1,4-dioxane. The homogeneous mixture was subjected to sonic waves in a sonicator for effective collision and distribution at 25°C for 1 h. The blends were cast on a glass plate and pre-treated with dichlorodimethylsilane. After drying at 60°C for 5 h, the blended samples were cured at 100, 150, 200 for 1 h each and 250°C for 2 h in an air oven. The red brown colored POSS reinforced CE (POSS-AECE) nanocomposites were obtained with thicknesses ranging from 0.3 to 0.5 mm (Scheme 2).

## Characterization

FT-IR spectra were recorded on a Perkin Elmer 6× FT-IR spectrometer. About 100 mg of optical-grade KBr was ground with sufficient quantity of the solid sample to make 1 wt% mixture for making KBr pellets. All ^1^H and ^13^C NMR analyses were done in CDCl_3_ and recorded on a Bruker 300 and Bruker 500 spectrometer.

The calorimetric analysis was performed on a Netzsch DSC-200 differential scanning calorimeter. Measurements were performed under a continuous flow of nitrogen (20 mL/min). All the samples (about 10 mg in weight) were heated from ambient temperature to 300°C and the thermograms were recorded at a heating rate of 10°C/min. Thermo gravimetric analysis (TGA) was performed on a Netzsch STA 409 thermo gravimetric analyzer. The samples (about 10 mg) were heated from ambient temperature to 700°C under a continuous flow of nitrogen (20 mL/min), at 10°C/min.

The dielectric constant of the neat AECE and the POSS-AECE systems were determined with the help of impedance analyser (Solartron impedance/gain phase analyser 1260) at room temperature using platinum (Pt) electrode at a frequency range of 1 MHz. This experiment was repeated four times at the same conditions.

A JEOL JSM-6360 field emission Scanning Electron Microscope (SEM) was used to record the SEM pictures and the samples were prepared by coating gold on their surface for SEM measurements. A JEOL JEM-3010 analytical transmission electron microscope, operating at 300 kV with a measured point-to-point resolution of 0.23 nm has been used to capture the micrograph of the composites. TEM samples were prepared by dissolving polymers in DMF mounted on carbon-coated Cu TEM grids and dried 24 h at RT to form a film in <100 nm size.

Water contact angle measurements were taken using a Ramehart Inc. goniometer. Contact angle measurements were taken with 5 μl of water and DI used as probe liquid.

## Results and discussion

**Figure 1:**
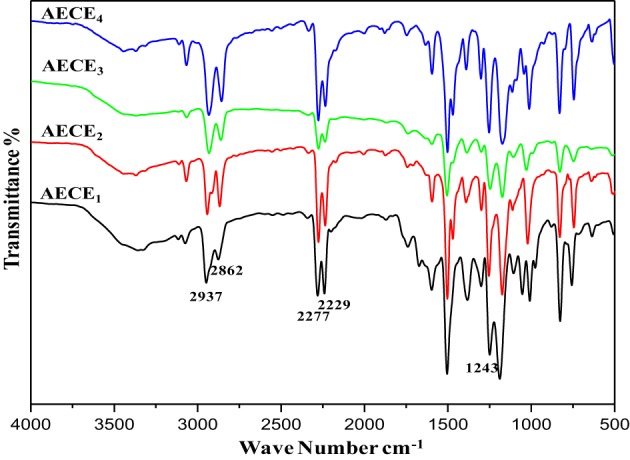
**FT-IR spectra of linear aliphatic alkoxy core bridged cyanate esters**.

The flexible linear aliphatic alkoxy core bridged bisphenol CE (Scheme 1) was synthesized with good yield, in four steps; in the first step, the synthesis of linear dialdehyde by o-arylation, using the respective dibromoalkane [(CH_2_)_*n*_ *n* = 2, 4, 6, and 8] and 4-hydroxy benzaldehyde in the presence of anhydrous sodium carbonate in DMF was done. In the second step, the linear dialdehyde was oxidized using MCPBA to get linear oxydialdehyde, and in the third step the hydrolysis of linear oxydialdehyde was carried out to get the respective ether linked diols. Finally, during the fourth step the cyanogenation of diols was done to get the respective linear ether linked cyanate esters. The molecular structure of all monomers was confirmed by the FT-IR (Figure 1). From FTIR spectra, the appearances of peak at 2229–2277 cm^−1^ for OCN and 2862, 2937 cm^−1^ correspond to symmetric and asymmetric stretching, respectively for aliphatic CH_2_ group. The ^1H and ^13^CNMR^ (Figures S1–S8 present in supporting information) further confirms the structure of AECE monomers.

**Figure 2:**
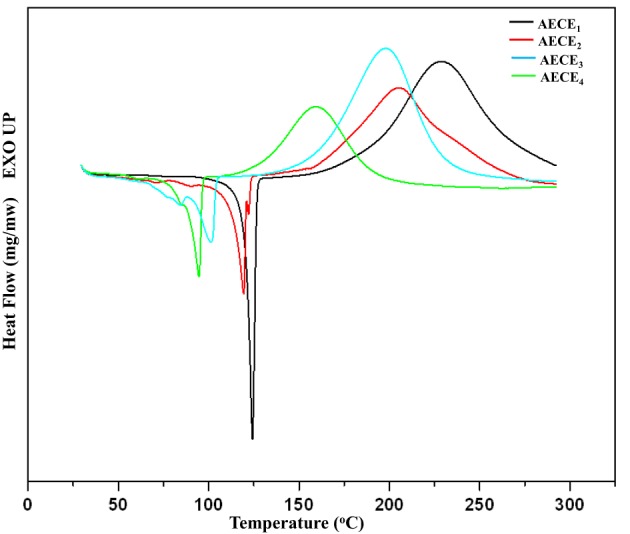
**Curing behaviors of cyanate ester monomers (AECE_1-4_)**.

One of the key disadvantages of conventional CE resins is their poor curing characteristic; that is, normally CE should be cured at a higher curing temperature to obtain the desired performance. Generally, higher curing temperatures result in higher internal stress and more defects in the cured resin, thereby decreasing the overall properties of the resultant resin. The curing behavior of the linear ether linked CE monomers was studied by the DSC analysis. The DSC thermogram of linear ether linked CE monomers is shown in Figure 2. The DSC thermogram shows a single exothermic peak which is attributed to the cyclotrimerization of CE. The onset of the curing of CE monomers commences at a lower temperature, with an increase in the chain length of the monomer. The −ΔH polymerization enthalpy values for AECE_1−4_ monomers are in the range between 567 mJ/mg and 355 mJ/mg. In the case of AECE_4_ monomer, the onset and the peak top temperatures are lower and the AECE_1_ monomer shows a higher onset and peak top temperature. In the case of the other monomers (AECE_2_, AECE_3_), the onset and peak top temperatures lie in between these two extremes, which are lower than that of conventional CE monomer (BADy) and the values are presented in Table 1. This can be explained by the presence of the electron donating ether linkage; the aliphatic ether chain length increases and may lead to an increase in the electron density, which decreases the curing temperature (Vengatesan et al., 2013).

**Table 1:** **Curing studies of AECE monomers**.

**Sample**	**T_i_ (°C)**	**T_p_ (°C)**	**T_f_ (°C)**	**ΔH polymerization mJ/mg**
AECE_1_	175	228	263	569
AECE_2_	163	205	251	512
AECE_3_	144	197	230	446
AECE_4_	120	161	186	355

T_i_, initial polymerization temperature; T_p_, peak curing temperature of the monomers; T_f_, final polymerization temperature.

**Figure 3:**
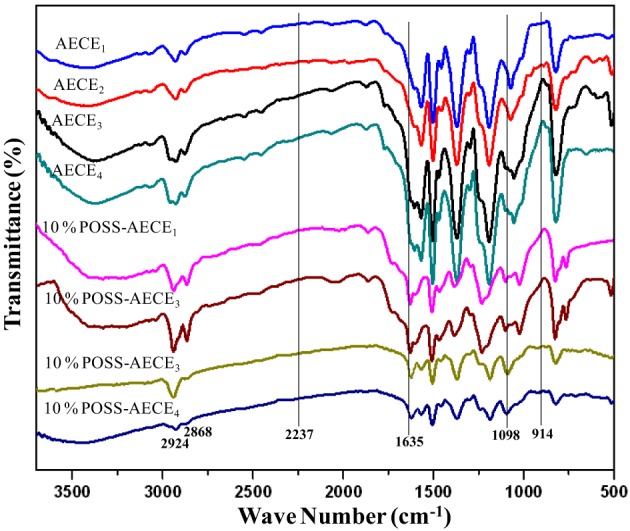
**FT-IR spectra of POSS-AECE nanocomposites**.

The POSS-AECE nanocomposites (Scheme 2) were prepared by the incorporation of varying weight percentages of OG-POSS (5 and 10 wt %) in to AECE resin. Figure 3 shows the FTIR spectra of varying combinations of POSS-AECE nanocomposites. The disappearance of the bands at 2237 cm^−1^ (the –OCN group of the CE) and 914 cm^−1^ (the glycidyl group of OG-POSS), and the appearance of a band at 1635 cm^−1^ corresponding to an oxazoline linkage, confirms the occurrence of a reaction between the OCN group of CE and glycidyl group of OG-POSS. The symmetric and asymmetric stretching frequencies of the -CH_2_ group appeared at 2924 and 2868 cm^−1^, respectively. The presence of a band at 1098 cm^−1^ due to Si-O-Si confirms the presence of a silica network in the hybrid.

The glass transition temperature (Netzsch DSC-200) of the neat AECEs and POSS–AECE nanocomposites are shown in Table 2. The POSS-AECE nanocomposites exhibit increased Tg compared to that of neat AECE and this may be explained by the following reasons. The incorporation of POSS increases the cross-linking density of the resulting nanocomposites, and increases the rigidity of the nanocomposites system. Also the increase in the values of Tg, is due to the restricted segmental motion imparted by the polymer chain with chemical interaction between the polymeric chain and POSS. The incorporation of POSS creates porosities (free volume) in the nanocomposites; this effect has, however, been counteracted by an increase in the cross-linking density. Among the POSS-AECE systems the AECE_1_ based system possesses higher values of Tg and the AECE_4_ based system exhibits the lowest value of Tg. The values of the other systems are lie in between these two systems. The Tg value of POSS-AECE decreased linearly with an increasing chain length. The flexible aliphatic backbone influences the glass transition temperature of the hybrid systems by increasing the internal rotations and thermal motion of the polymer chains, which in turn decreases the values of Tg.

**Table 2:** **Thermal and dielectric properties of neat AECE and POSS-AECE nanocomposites**.

**Experiment**	**T_g_ (°C)**	**10% weight loss (°C)**	**50% weight loss (°C)**	**Char yield (at 900°C) (%)**	**Dielectric constant (έ)**
AECE_1_	220	336	417	20.1	4.2
5%POSS-AECE_1_	228	351	425	23.5	3.6
10%POSS-AECE_1_	235	375	430	27.2	3.1
AECE_2_	217	330	423	16.2	3.8
5%POSS-AECE_2_	222	329	427	19.0	3.5
10%POSS-AECE_2_	230	331	429	21.4	2.9
AECE_3_	210	328	410	10.2	3.6
5%POSS-AECE_3_	223	325	420	15.1	3.0
10%POSS-AECE_3_	228	321	427	19.4	2.6
AECE_4_	203	336	420	5.1	3.2
5%POSS-AECE_4_	211	338	425	14.8	2.9
10%POSS-AECE_4_	221	341	440	16.0	2.4

**Figure 4:**
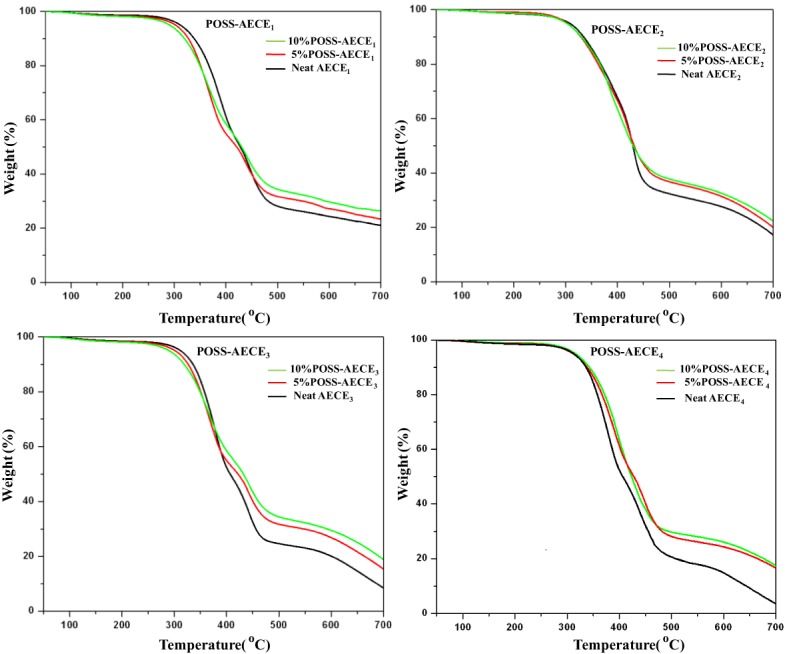
**TGA thermogram of POSS-AECE nanocomposites**.

Thermo gravimetric analysis (Netzsch STA 409) was used to ascertain the thermal stability of neat AECE and OG-POSS reinforced AECE nanocomposites, and the data obtained from the TGA are presented in Figure 4 and Table 2. The POSS-AECE nanocomposites exhibited higher thermal stability and higher char yield than those of neat AECE resin. Generally, the improvement in the thermal stability of hybrid composites is related to the degree of the interaction between the polymer matrix and the inorganic phase, and to the fact that the inorganic component has an inherently good thermal stability. In addition, the presence of the rigid cubic siloxane and the partial ionic nature of the inorganic Si-O-Si skeleton contributed to the thermal stability of the hybrid nanocomposites. The POSS core gave an additional heat capacity which stabilizes the materials against the thermal decomposition. The loss of organic materials from the segmental decomposition through gaseous fragments could be reduced by the well dispersed POSS core in the AECE matrix. Further, it was also observed that the incorporation of OG-POSS into the AECE resin reduced both the volatile decomposition and the polymer flammability. Further, when POSS degrades, it leaves an inert silica layer which can form a protective layer on the surface of the material preventing further oxidation of the inner part of the matrix. Among the four nanocomposites (AECE_1−4_), the AECE_1_ based POSS incorporated nanocomposites possesses higher thermal stability, while that of AECE_4_ nanocomposites exhibits the lowest thermal stability due to the higher chain length, which induces free rotational movement.

A lower dielectric constant is one of the most desirable properties for next generation microelectronic devices. Table 1 gives the values of the dielectric constant (Solartron impedance/gain phase analyzer 1260) of POSS-AECE nanocomposites with varying percentage of POSS concentration. The dielectric constant of the hybrid decreases with an increase in the amount of POSS. The reduction in the value of dielectric constant of POSS-AECE hybrids is due to the creation of pores and enhanced free volume contributed by the rigid and bulky core structured POSS molecule (Wu et al., 2007). The less polar nature of Si-O-Si linkage and aliphatic flexible group in the POSS-AECE system reduces the value of dielectric constant. The signal propagation delay time of the integrated circuits is proportional to the square root of the dielectric constant of the matrix, and the signal propagation loss is proportional to the square root of the dielectric constant and dissipation factor of the matrix. Thus, a material with a low dielectric constant and low dissipation factor will reduce the signal propagation delay time and the signal propagation loss (Nagendiran et al., 2010), and can be used to improve the effective and efficient functioning of electronic instruments. Among the POSS-AECE systems studied, the AECE_4_ based system possesses the lowest value of dielectric constant and AECE_1_ based system exhibits the highest value of dielectric constant. The values of the other two systems are lie in between these two extremes. This may be explained by the presence of higher aliphatic chain which reduces the polarity and enhances the free rotation of the molecules.

**Figure 5:**
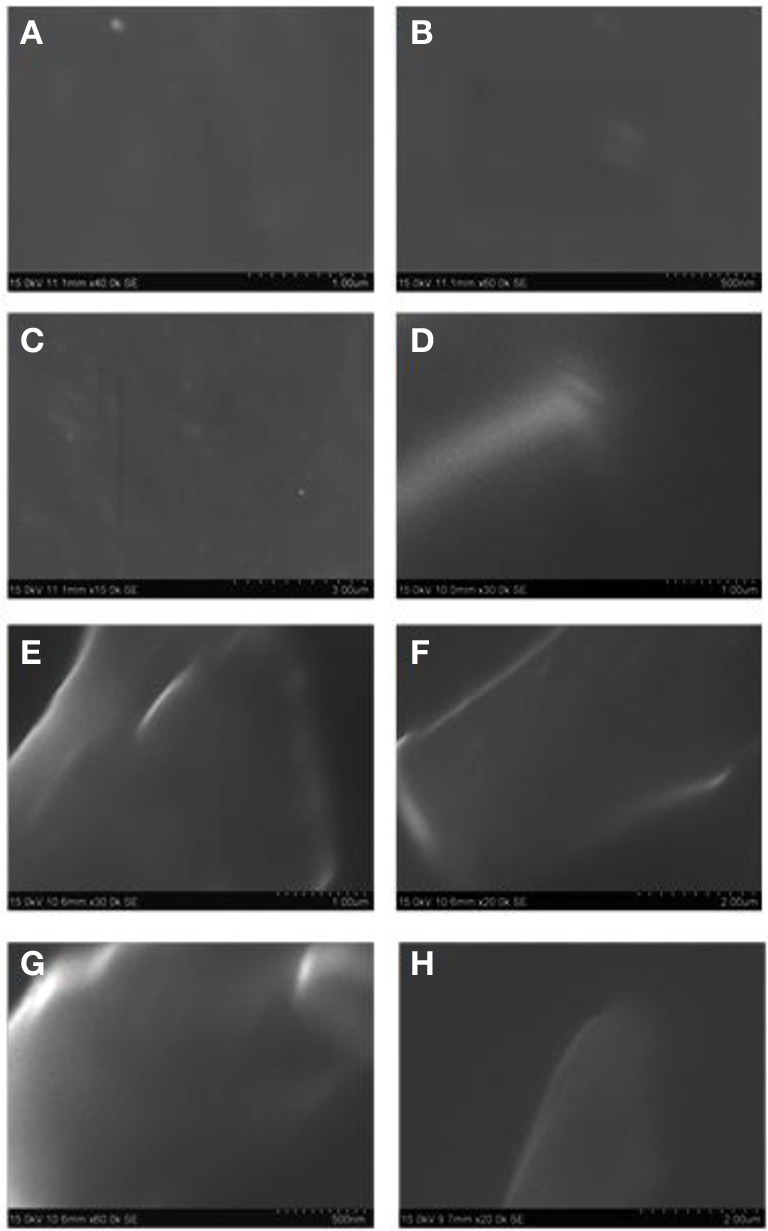
**SEM micrograph of (A)**. Neat AECE_1_, **(B)**. 10% POSS-AECE_1_, **(C)**. AECE_2_, **(D)**. 10% POSS-AECE_2_, **(E)**. Neat AECE_3_, **(F)**. 10% POSS-AECE_3_, **(G)**. Neat AECE_4_ and **(H)**. 10% POSS-AECE_4_.

The SEM (JEOL JSM-6360) is used to investigate the morphology of neat CE and OG-POSS reinforced AECE nanocomposites. Figure 5 shows the SEM micrograph of neat AECE and POSS-AECE nanocomposites. The SEM images indicated the smooth and homogeneous morphology of the neat AECE and POSS-AECE systems. The POSS incorporated system exhibited a featureless morphology and no discernable phase separation was observed. This indicates the good miscibility (compatibility) of POSS with AECE and also confirms the molecular level dispersion of POSS in the AECE nanocomposites. The homogeneous morphology could be ascribed to the formation of covalent bonding between POSS and the CE. This was further confirmed by the TEM analysis.

**Figure 6:**
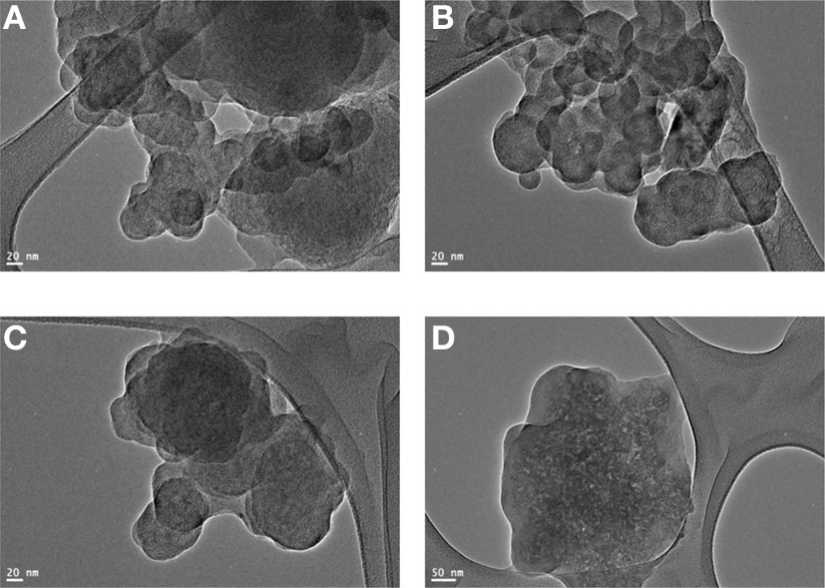
**TEM image of (A)**. 10% POSS-AECE_1_, **(B)**. 10% POSS-AECE_2_, **(C)**. 10% POSS-AECE_3_ and **(D)**. 10% POSS-AECE_4_.

Figures 6A–D represents the TEM micrographs (JEOL JEM-3010) of 10 wt% of OG-POSS reinforced AECE nanocomposites. It shows the homogeneous morphology, and no localized domains were observed. Only a few darker points were observed at approximately about 50 nm in the polymer matrix, which represents the dispersion of the OG-POSS in the AECE matrix. This observation indicates that the nanocomposite is a material with a particle size of the dispersed phase having at least one dimension of less than 100 nm (Komarneni, 1992). Thus, the POSS moieties are well dispersed at a nanometer scale in the AECE matrix to form a POSS-AECE network.

The hydrophobicity of the neat AECEs and POSS incorporated POSS-AECEs was determined from the contact angle measurements using the goniometer (GBX, France). Contact angle measurements were taken with water and diiodomethane, to determine how POSS influences the hydrophobic nature of the CE matrix. The values of contact angle of neat AECE and POSS incorporated AECEs are presented in Table 3. With an incorporation of 5 and 10 wt% OG-POSS, the values of the contact angles increased, which indicates that the presence of POSS in the AECE matrix improves the hydrophobicity of the nanocomposite surfaces. The increase in hydrophobicity is mainly ascribed to the covalent interaction between POSS and AECEs, and also the less polar nature of the Si-O-Si linkage in the POSS, which leads to an increase in the hydrophobic nature of the AECE matrices (Tang and Lewin, 2009; Wang et al., 2011). Among the four systems the AECE_4_ based POSS incorporated system possesses the highest value of water contact angle, and the AECE_1_ based POSS incorporated system exhibits the lowest value of the water contact angle. This may be explained due to the higher aliphatic flexible linear alkoxy chain that reduces the polarity, and increases the hydrophobic nature of the resulting hybrid system compared to that of the other three systems.

**Table 3:** **Contact angle and surface free energy of neat AECE and POSS-AECE nanocomposites**.

**Experiments**	**Contac angle (θ)**		**Surface free energy**		
	**Water**	**Diiodomethane**	**γ *^d^***	**γ *^p^***	**Γ**
AECE_1_	85	48	35.4	2.7	38.1
5% POSS-AECE_1_	94	60	28.6	1.6	30.1
10% POSS-AECE_1_	103	67	24.6	0.5	25.1
AECE_2_	88	52	33.2	2.0	35.2
5% POSS-AECE_2_	98	63	26.8	1.0	27.8
10% POSS-AECE_2_	107	70	22.9	0.2	23.1
AECE_3_	93	58	29.7	1.6	31.3
5% POSS-AECE_3_	104	68	24.0	0.4	24.4
10% POSS-AECE_3_	112	75	20.1	0.1	20.2
AECE_4_	98	59	29.2	0.9	30.0
5% POSS-AECE_4_	109	71	22.3	0.1	22.4
10% POSS-AECE_4_	121	77	19.1	0.1	19.2

Two approaches are employed to calculate the surface energy of a solid material: the geometric mean method (Wang et al., 2011), and three liquid method (Xiang and Chung, 2001). These two methods are the most widely used for surface energy analyses.

In this work we used the geometric mean method estimates the surface energy of a solid (γ_*S*_) as follows:

(1) $$\text{Cos}\theta \text{=2/}\gamma_{L}\left[{\left(\gamma_{L}^{d}\cdot \gamma_{S}^{d}\right)}^{1/2}+{\left(\gamma_{L}^{p}\cdot \gamma_{S}^{p}\right)}^{1/2}\right]-1$$

where γ_*L*_, γ^*d*^_*L*_, and γ^*p*^_*L*_ are the surface energies of the test liquid and γ^*d*^_*S*_ and γ^*p*^_*S*_ can be calculated from the measured value of contact angles (θ). The *d* refers to the London dispersion forces and *p* refers to the polar forces, including all the interactions established between the solid and liquid, such as Keesom dipole-dipole, Debye dipole-induced dipole, and hydrogen bonding, etc. A low surface free energy is important in many practical applications. Considerable effort has been focused on the development of non-wettable, low surface free energy polymeric materials with film forming characteristics. The thrust for those activities is provided by commercial applications in aerospace, lithography, clothing, wetting, dyeing, intergraded sensors, and protection against biological and other fouling. In general, amorphous, comb like polymers possessing a flexible linear backbone onto which side-chains with low intermolecular interactions are attached, exhibit low surface free energy values (Tsibouklis and Nevell, 2003).

The surface free energy values of neat AECEs and POSS-AECEs are listed in Table 3. From the Table it can be seen that the surface free energy value decreases with an increase in the percentage content of POSS incorporated in to AECE matrices. This is due to the flexible aliphatic alkoxy core that reduces the polarity of the system; also, the less polar Si-O-Si linkage in the POSS covalently incorporated into the AECE matrix, which reduces the value of the surface free energy of the resulting composite system (Tang and Lewin, 2009; Wang et al., 2011). Among the four systems, the AECE_4_ based system exhibited the lowest values of surface free energy when compared to that of other three hybrid systems and this is due to the presence of higher aliphatic flexible linear alkoxy chain which reduces the polarity of the resulting composite systems.

## Conclusion

A new series of linear aliphatic alkoxy core bridged bisphenol CE based POSS-AECE hybrid nanocomposites have been developed. The formation of nanocomposites was confirmed by the FT-IR. Data from the thermal analysis indicate that POSS-AECE nanocomposites exhibit excellent thermal properties, such as higher Tg, thermal stability and higher char yield, than those of neat AECE. The values of the dielectric constant (έ) of the POSS incorporated AECE are lower than those of neat AECE. From the contact angle studies it is inferred that the POSS incorporated AECE system possesses better hydrophobicity and lower surface free energy, when compared to those of neat AECE.

### Conflict of interest statement

The authors thank BRNS, G. No: 2012/37C/9/BRNS, Mumbai, Govt. of India., for the financial support and Dr. Manmohan Kumar, Senior Scientific Officer, BARC, Mumbai for his support.
