# Supplementary material for: Low dielectric and low surface free energy flexible linear aliphatic alkoxy core bridged bisphenol cyanate ester based POSS nanocomposites
Source: Front Chem. 2013 Oct 14;1:19. doi: 10.3389/fchem.2013.00019 (PMC3982528; doi:10.3389/fchem.2013.00019)
Supplement: Supplementary file 1 [file Presentation1.PDF]

**Low dielectric and low surface free energy flexible linear aliphatic alkoxy core bridged bisphenol cyanate ester based POSS nanocomposites**

**S. Devaraju[a,b], P.Prabunathan[a], M. Selvi[a] and M. Alagar[a*]**

*[a]Polymer Composites Lab, Department of Chemical Engineering, A.C.Tech, Anna University, Chennai - 600 025, India.

[b]Next MEMS lab, School of Mechanical Engineering, Pusan National University, Busan 609-735, South Korea.

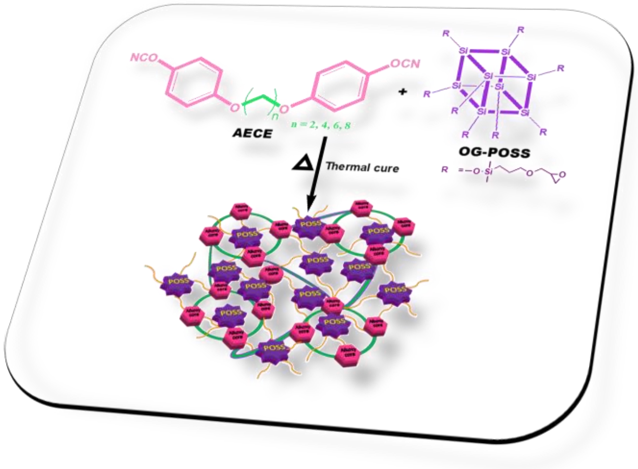


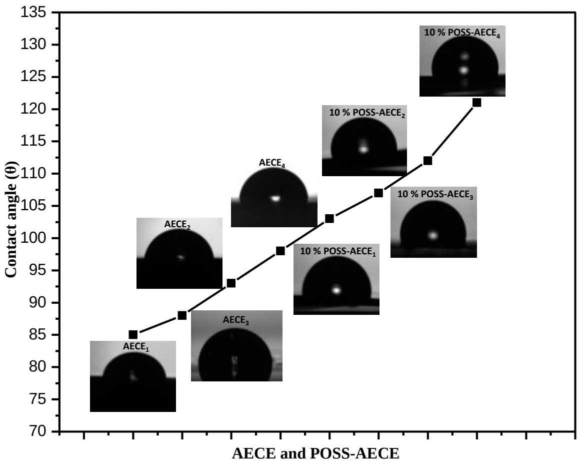


- Develop new type of flexible linear aliphatic alkoxy core bridged bisphenol cyanate ester (AECE) based POSS nanocomposites.
- POSS reinforced system exhibits higher thermal stability and low k (k=2.4)
- POSS reinforced system possesses better hydrophobic in nature and low surface free energy ($\gamma$ = 19.1)
