# Supplementary material for: Low dielectric and low surface free energy flexible linear aliphatic alkoxy core bridged bisphenol cyanate ester based POSS nanocomposites
Source: Front Chem. 2013 Oct 14;1:19. doi: 10.3389/fchem.2013.00019 (PMC3982528; doi:10.3389/fchem.2013.00019)
Supplement: Supplementary file 2 [file DataSheet1.PDF]

Low dielectric and low surface free energy flexible linear aliphatic alkoxy core bridged bisphenol cyanate ester based POSS nanocomposites

S. Devaraju[a,b], P.Prabunathan[a], M. Selvi[a] and M. Alagar [a*]

*[a]Polymer Composites Lab, Department of Chemical Engineering, Alagappa College of Technology, Anna University, Chennai - 600 025, India.

[b]Next MEMS lab, School of Mechanical Engineering, Pusan National University, Busan 609-735, South Korea.

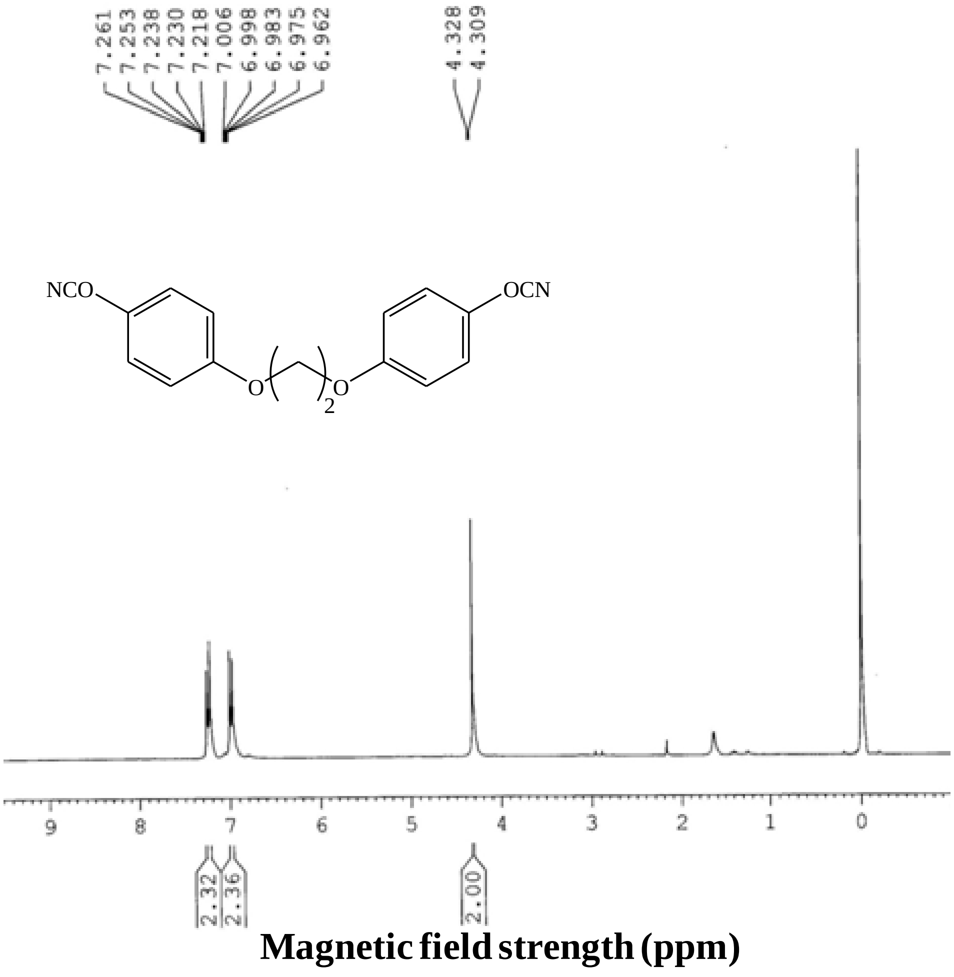

Figure S1: ^{1}HNMR Spectrum of AECE$_1$

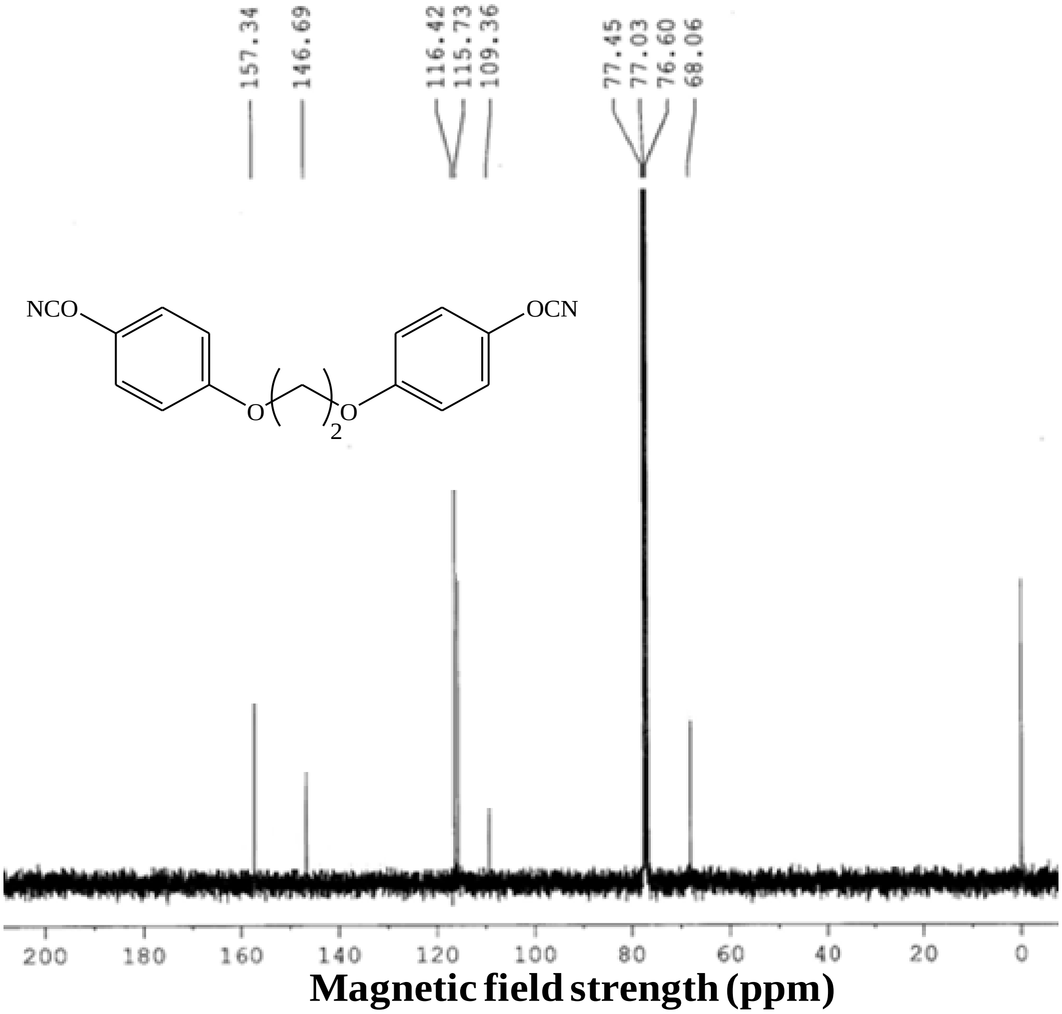

Figure S2: ^{13}CNMR Spectrum of $AECE_1$

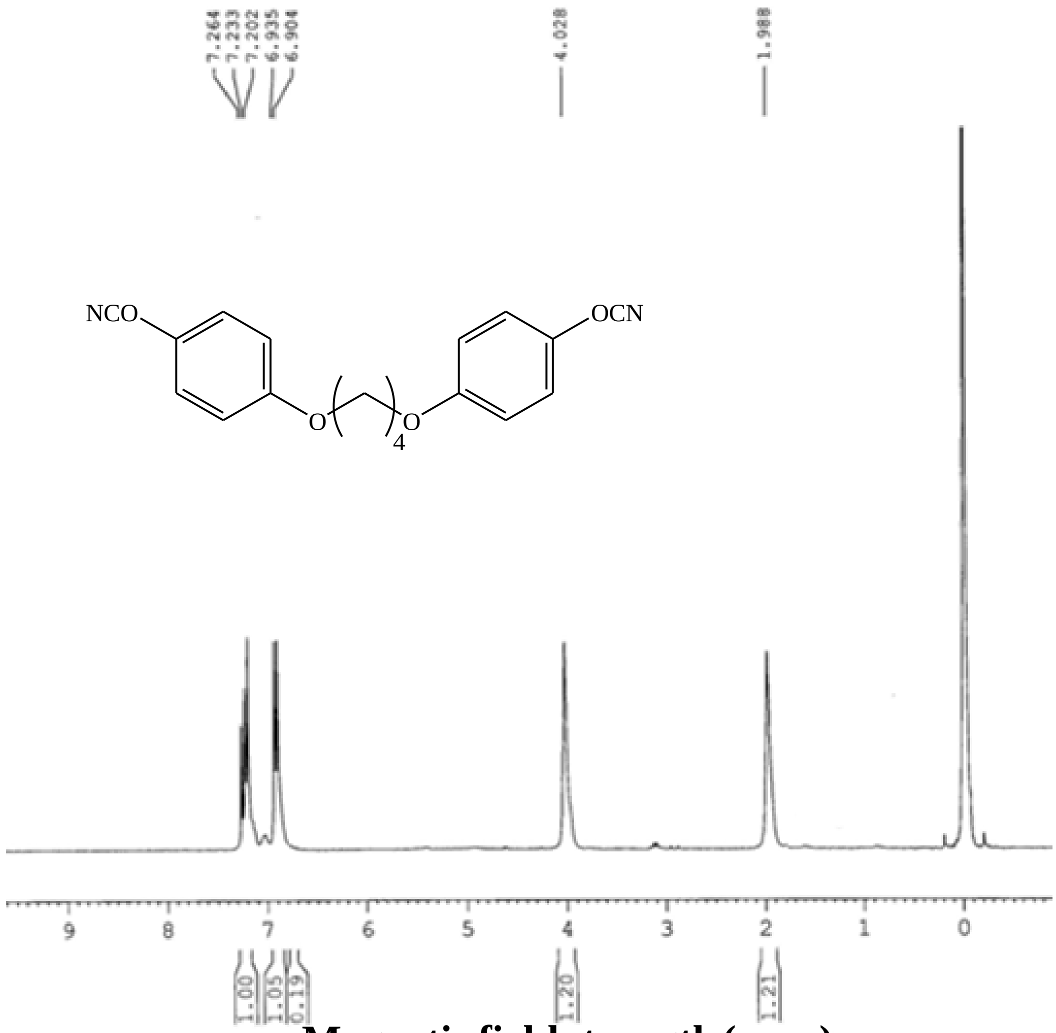

Magnetic field strength (ppm)

Figure S3: ^{1}HNMR Spectrum of AECE$_2$

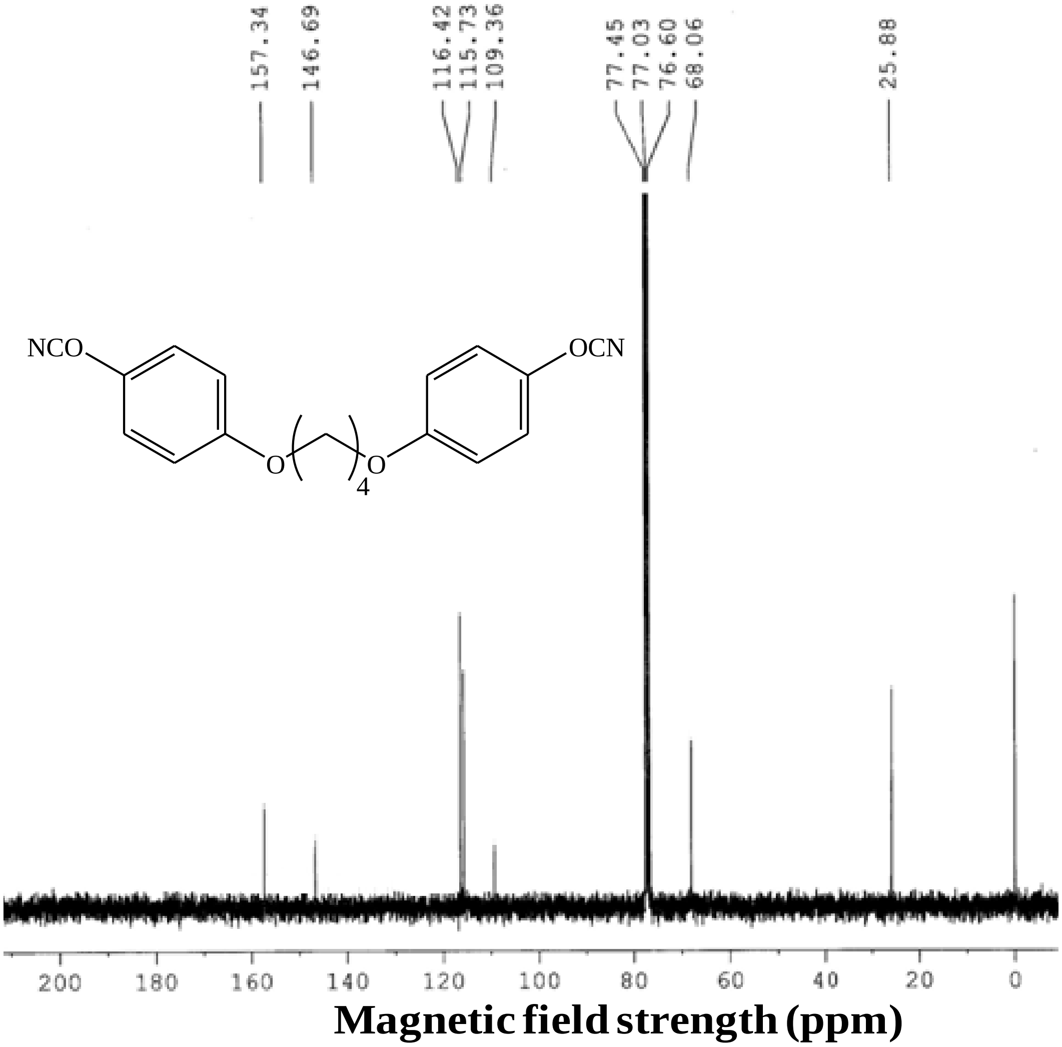

Figure S4: ^{13}CNMR Spectrum of $AECE_2$

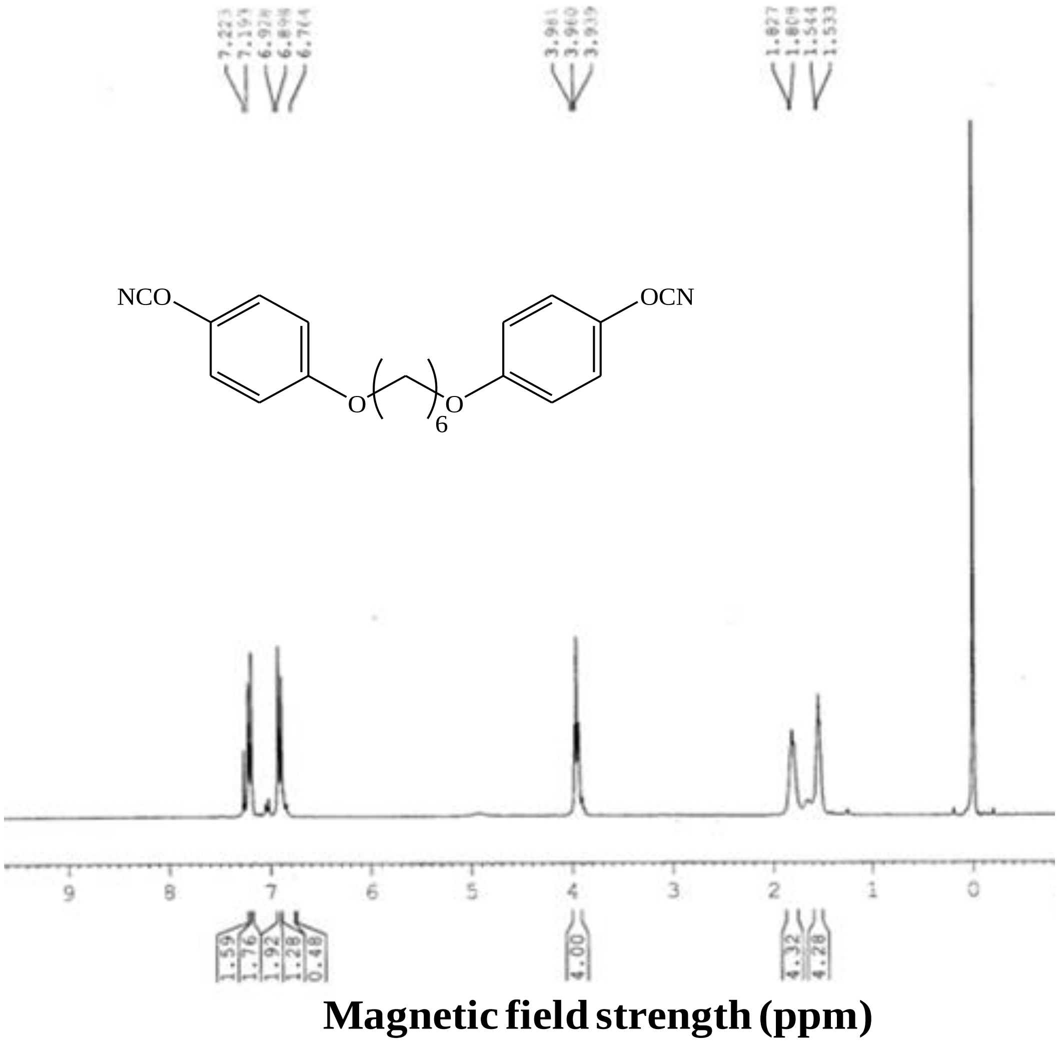

Figure S5: ^{1}HNMR Spectrum of $AECE_3$

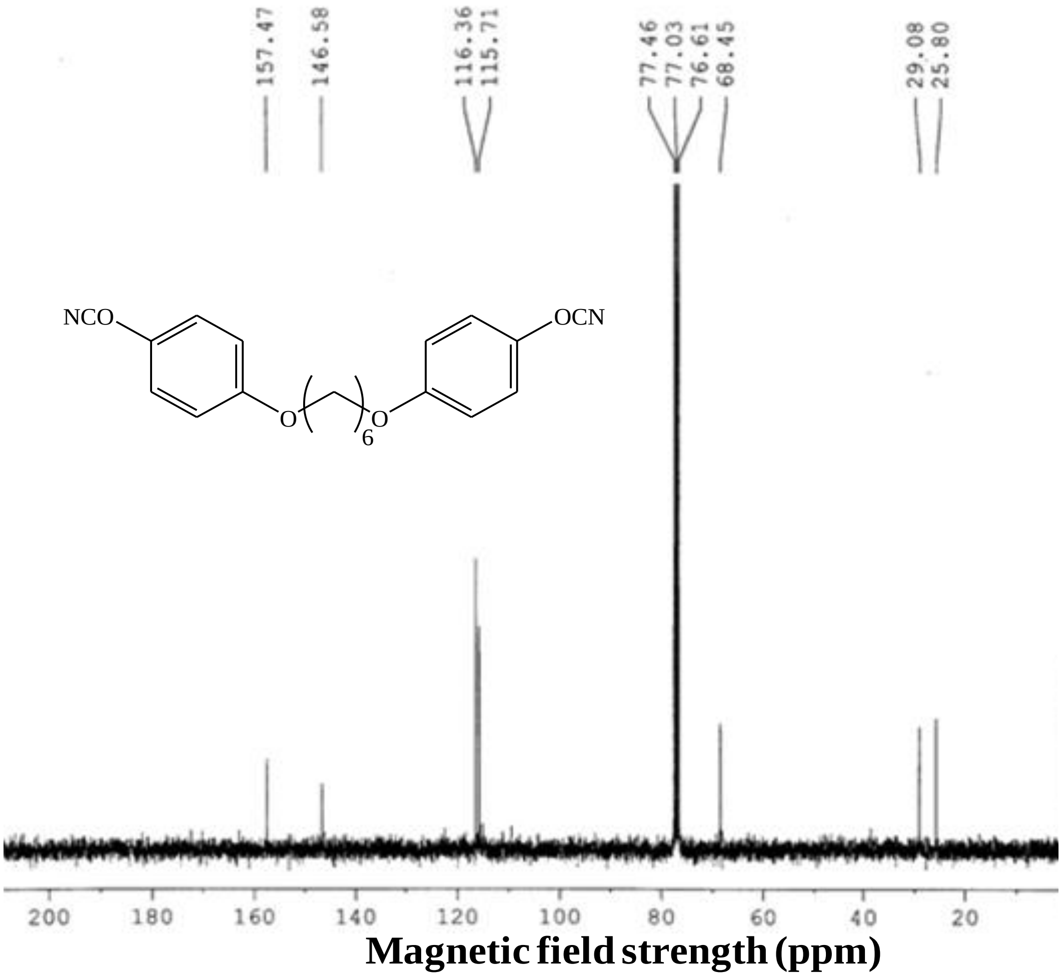

Figure S6: ^{13}CNMR Spectrum of $AECE_3$

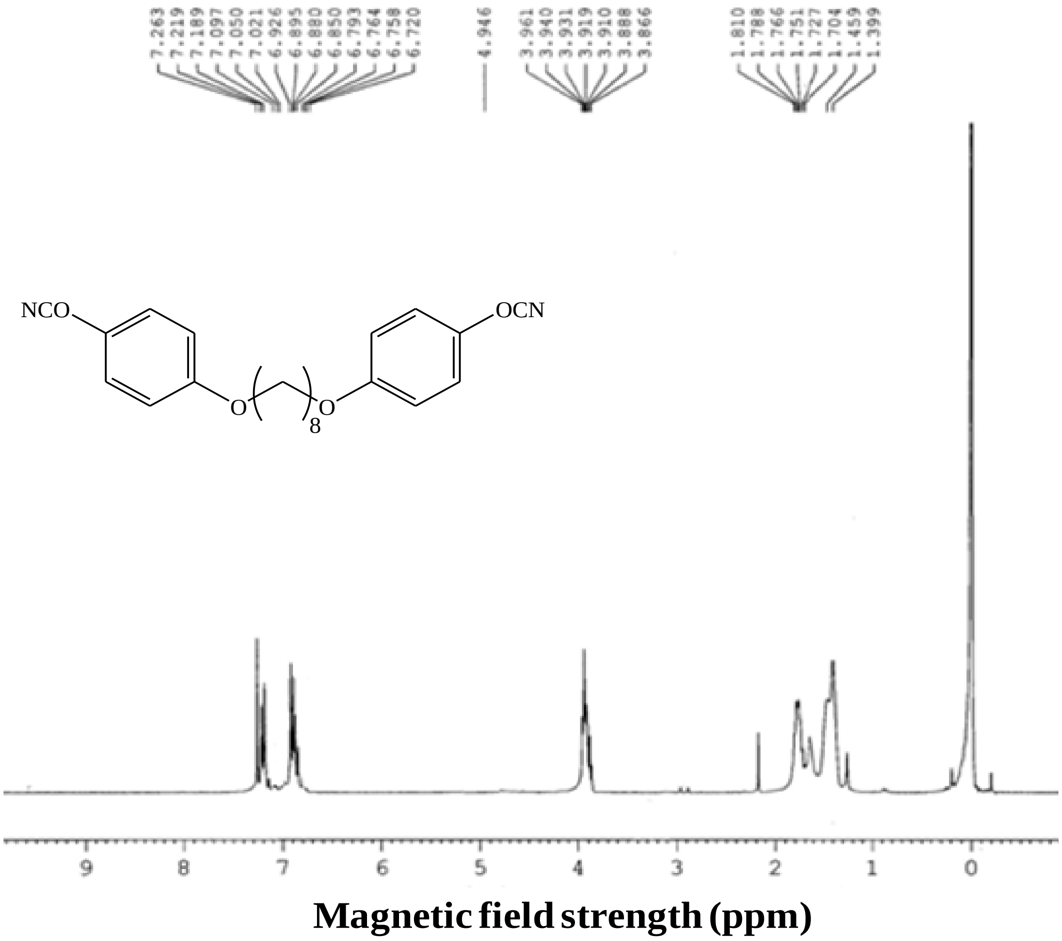

Figure S7: ^{1}HNMR Spectrum of $AECE_4$

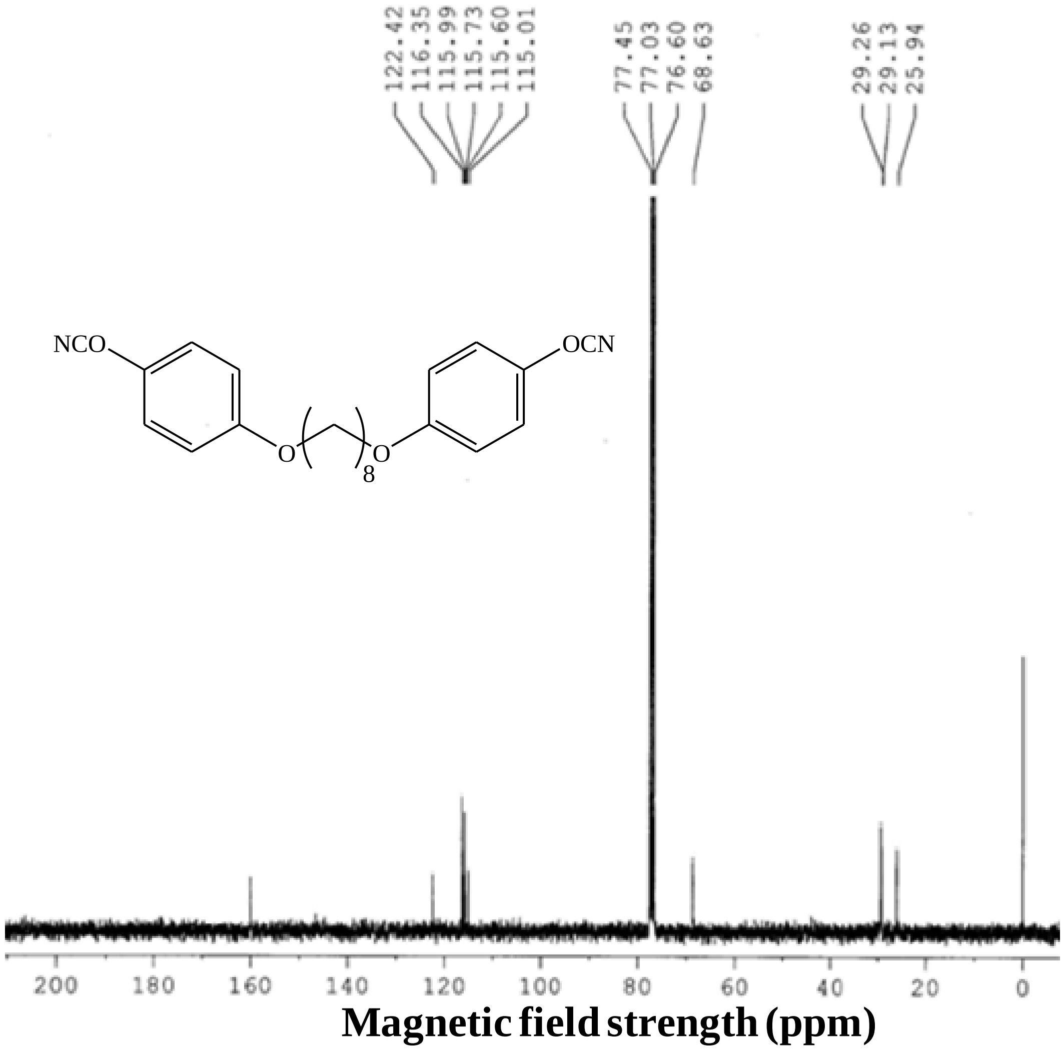

Figure S8: ^{13}CNMR Spectrum of AECE$_4$
